# Macrophages as a Source and Recipient of Wnt Signals

**DOI:** 10.3389/fimmu.2019.01813

**Published:** 2019-07-31

**Authors:** Elizabeth S. Malsin, Seokjo Kim, Anna P. Lam, Cara J. Gottardi

**Affiliations:** Department of Pulmonary Medicine, Feinberg School of Medicine, Northwestern University, Chicago, IL, United States

**Keywords:** macrophages, monocytes, beta catenin, Wnt signaling, immunity

## Abstract

Macrophages are often viewed through the lens of their core functions, but recent transcriptomic studies reveal them to be largely distinct across tissue types. While these differences appear to be shaped by their local environment, the key signals that drive these transcriptional differences remain unclear. Since Wnt signaling plays established roles in cell fate decisions, and tissue patterning during development and tissue repair after injury, we consider evidence that Wnt signals both target and are affected by macrophage functions. We propose that the Wnt gradients present in developing and adult tissues effectively shape macrophage fates and phenotypes. We also highlight evidence that macrophages, through an ability to dispatch Wnt signals, may couple tissue debridement and matrix remodeling with stem cell activation and tissue repair.

## Introduction

**Figure 1:**
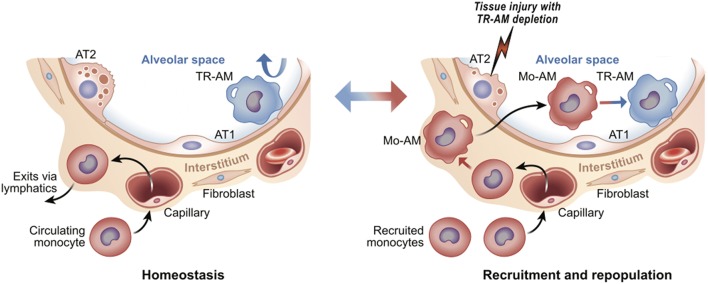
Tissue resident vs. recruited macrophages. Using lung as an example, tissue resident alveolar macrophages (TR-AM) self-renew to maintain surfactant homeostasis; monocytes are not recruited from the circulation **(Left)**. After injury, TR-AMs are depleted and ultimately replaced by circulating monocytes (monocyte-derived alveolar macrophages, or Mo-AM) **(Right)**. Upon injury, Mo-AMs are transcriptionally distinct from TR-AMs. Over time, Mo-AMs become transcriptionally indistinguishable from TR-AMs (7). The alveolar airway surface epithelium is lined by surfactant producing Alveolar Type 2 (AT2) and gas-exchange promoting Type 1 cells (AT1).

Macrophages are present in virtually every tissue, playing crucial roles in homeostatic tissue maintenance and coordinating cellular responses to tissue injury [reviewed in (1, 2)]. Most tissues in the steady state contain diverse populations of so-called “tissue-resident” macrophages. These tissue-resident macrophages may differ in their ontogeny, ability to proliferate, and specific micro-niches within the organ. For example, some macrophages populate organs during early embryogenesis (e.g., microglia) or the early postnatal period (e.g., alveolar macrophages in the lung). These macrophages maintain their population via proliferation *in situ*, thus keeping the tissue micro-niche “occupied” and apparently “closed” to circulating monocytes (3). Circulating monocytes can enter and patrol these closed tissues, but without appropriate differentiation stimuli, fail to differentiate into macrophages, and instead exit via lymphatics (4). In contrast, tissue-resident macrophages in other tissues (e.g., gut, skin and heart) are relatively short-lived and require constant influx and differentiation of monocytes to maintain their population (5, 6). Regardless of these differences, once tissue-resident macrophages are depleted, such as after ionizing radiation or in response to tissue injury, circulating monocytes enter the tissue and differentiate into macrophages to repopulate the “open” niche. The fate of these recruited, monocyte-derived macrophages depends on both the tissue, as well as the type and extent of injury. These macrophages can disappear after injury resolution or adapt to the empty niche and become long-living resident cells by taking on epigenetic, transcriptomic, and functional features of tissue-resident macrophages seeded during development [reviewed in (3)] (Figure 1). Thus, while the local tissue environment is likely critical in shaping macrophage identity and functionality, the specific signals and transcriptional programs they direct remain poorly defined.

**Figure 2:**
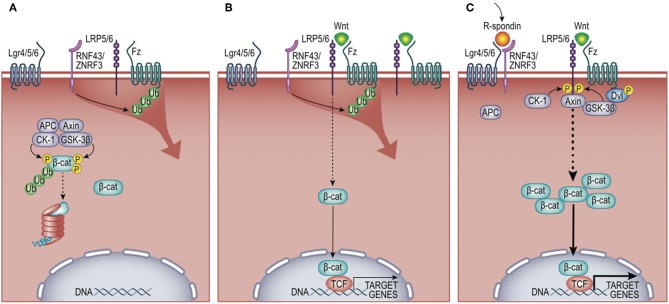
Wnt/β-cat signaling pathway. **(A)** In the absence of Wnt ligand, cytosolic β-cat is constitutively flagged for degradation by multi-protein complex comprising Adenomatosis Polyposis Coli (APC) protein, Axin, Casein Kinase 1 (CK-1), and Glycogen Synthase Kinase-3 beta (GSK-3β). **(B)** Wnt engagement of Frizzled (Fz) and low-density lipoprotein receptor-related protein 5 or 6 (Lrp5/6) inhibits β-cat turnover, favoring nuclear translocation and activation of target genes, including the negative feedback regulators zinc and ring finger proteins RNF43/ZNRF3. These E3 ligases antagonize Wnt signals by ubiquitylating Fz receptors, promoting their destruction. **(C)** R-spondins synergistically promote Wnt signals by binding leucine-rich repeat-containing G-protein coupled receptors (LGR4/5/6) and the E3 ligases RNF43/ZNRF3. This limits the ubiquitylation of Fz receptors, permitting enhanced activation of β-cat target genes. Non-canonical (i.e., β-cat-independent) Wnt signaling is not shown and described elsewhere (14).

Wnt signaling regulates many developmental processes, including cell fate decisions, migration, and the overall spatial patterning of tissues (8). It is one of a number of modular pathway “tools” in the developmental “toolkit” that drives tissue organization, where its contribution to immune biology is just emerging (9–11). The Wnt pathway is organized like many cell-to-cell signaling pathways that induce both transcriptional and behavioral responses. An extracellular Wnt ligand is released by a secretor-cell and received by a responder-cell through a receptor complex generally comprising Frizzled and LRP5/6 family members (12). Depending on the presence or absence of additional co-receptors (elaborated below), Wnt-signaling can activate gene expression via β-catenin (β-cat) paired with a DNA-binding factor from the T cell factor/Lymphocyte enhancer factor (TCF/Lef)-family (13) (Figure 2). This transcriptional mode of Wnt signaling is often referred to as “canonical” signaling, as it was first and remains the most thoroughly defined consequence of Wnt signaling. Wnt receptor activation can alternatively direct diverse cell responses (e.g., planar polarization and cytoskeletal organization) through a number of less defined pathways that are independent of, and often inhibitory to, β-cat transcriptional function, referred to as the “non-canonical” pathway (14, 15). The extent to which a Wnt signal triggered β-cat-dependent vs. -independent responses was historically thought to be due to the particular form of Wnt ligand expressed, where most Wnts (e.g., Wnt3a, Wnt1) activate β-cat, while a smaller subset (e.g., Wnt5a, Wnt11, Wnt4) often antagonize β-cat signaling and/or signal independently of β-cat (16). However, it is now clear that it is the combination of Wnts, receptors, and co-receptors that dictate whether a response will activate β-cat or not (12, 17). Thus, an overriding challenge of understanding Wnt signaling in tissue biology is its complexity. In humans, there are 19 Wnts, 10 Frizzled receptors, a number of co-receptors (Lrp5/6, Lgr4/5/6, Ror1/2, and Derailed/Ryk), as well as secreted factors that both antagonize (e.g., DKKs, Sfrps) or agonize (e.g., R-spondins) Wnt/receptor signaling [reviewed in (18)]. Adding further to complexity, Wnt signaling is used reiteratively by cells and their descendants as tissue development unfolds, such that the target genes activated by Wnts are cell-type and context-dependent (19). It is in this latter context that we focus this review on evidence that macrophages are both a source and recipient of Wnt signals, particularly regarding core macrophage functions. As Wnt ligands are lipid modified (20–22), restricting their solubility, and localized signaling (23), we speculate that short-range Wnt gradients present in developing and adult tissues may play key roles in shaping organ-specific macrophage functions. All Wnt-macrophage studies discussed below are also summarized in Table 1.

**Table 1:** Specific Wnt ligand studies mentioned in the text, listed numerically, with elucidated role in macrophage biology.

**Wnt ligand**	**Role in macrophage biology**	**Canonical or non-canonical pathway**	**Reference(s)**
Wnt3a	Increased expression in macrophages by hepatocyte debris engulfment in setting of injury	Canonical	(24)
	Released by macrophages in presence of Notch signaling from mammary stem cells	Unclassified	(25)
	Promotes macrophage proliferation	Canonical	(26)
	Induces macrophage polarization synergistically with IL-4 or TGFβ	Canonical	(27)
	Proinflammatory in microglia	Canonical	(28)
	Stimulates anti-inflammatory tumor-associated macrophages in setting of glioblastoma	Canonical	(29)
	Inhibits TNF-production in murine macrophages infected with *M. tuberculosis*	Canonical	(30)
	Limits migration of monocytes through cultured endothelial cells	Unclassified	(31)
	Promotes macrophage polarization when released by hepatic tumor cells	Canonical	(32)
Wnt4	Upregulated in lung macrophages to promote epithelial proliferation and repair post injury	Unclassified	(33)
Wnt5a	Induces alternative activation of macrophages resulting in tolerogenesis in sepsis and breast cancer	Non-canonical	(34)
	Bridges innate/adaptive immunity of stimulated human macrophages/T-cells in setting of mycobacterial infection	Unclassified	(35)
	Diminishes formation of macrophages from hematopoietic progenitors	Unclassified	(36)
	Stimulates macrophage phagocytosis, secretion of proinflammatory cytokines	Non-canonical	(37)
	Monocyte-derived Wnt5a drives inflammatory lymphangiogenesis in the retina	Unclassified	(38)
Wnt7a	Induces a monocyte-derived macrophage phenotypes that is pro-inflammatory with an alternative cytokine profiled and reduced phagocytic capacity	Unclassified	(39)
Wnt7b	Released during apoptosis of dermal macrophages to drive hair follicle activation	Unclassified	(40)
	Released from macrophages in setting of renal injury, promotes repair	Canonical	(41)
	Tumor associated macrophages promote breast cancer growth by secreting to promote angiogenesis	Canonical	(42)
Wnt10a	Released during apoptosis of dermal macrophages, drives hair follicle activation	Canonical	(40)
	Released by macrophages in presence of Notch signaling from mammary stem cells	Unclassified	(25)
Wnt11	Diminishes formation of macrophages from hematopoetic progenitors, silencing favors formation of macrophages	Unclassified	(36)
Wnt16	Released by macrophages in presence of Notch signaling from mammary stem cells	Unclassified	(25)
	Upregulated in lung macrophages to promote epithelial proliferation and repair post injury	Unclassified	(33)
WntD (*Drosophila*)	Increases macrophage resistance to extracellular pathogens, but susceptibility to intracellular pathogens	Non-canonical	(43)

*Given the known complexity and overlap of Wnt ligands and their signaling pathways, each study is also designated as having identified the downstream signaling pathway as canonical, non-canonical, or not classified*.

## Wnt Signaling in Macrophage Differentiation and Maintenance

### β-Catenin Signaling Directs Macrophage Differentiation

As discussed above, macrophage differentiation is highly contextual. Tissue-resident macrophages originate from embryonic or immediate post-natal tissues, where their transcriptional profile is shaped by their local environment [reviewed in (44)]. Conversely, monocytes arise from bone marrow progenitors throughout lifespan and, in response to tissue injury, become macrophages only after entry into an organ's interstitium. This spatial and environmental complexity makes studying of the pathways involved in monocyte development difficult, especially *ex vivo*. Nonetheless, *in vitro* studies suggest that β-cat signaling may promote monocyte differentiation from myeloblasts (45). Myeloblasts are a hematopoietic progenitor that also gives rise to the granulocytic series of blood cell types (e.g., neutrophils, basophils, eosinophils), where granulocyte-macrophage colony stimulating factor (GM-CSF) followed by macrophage colony stimulating factor (M-CSF) promote the production of myeloid cells and are essential for monocyte differentiation [reviewed in (46)]. Using this *in vitro* system, GM-CSF could direct a β-catenin/T-Cell Factor (β-cat/TCF) transcription program to specify the monocyte lineage. Careful mutational analyses of the beta-subunit of the GM-CSF receptor (GM-CSF-R) revealed molecular strategies that could enhance monocyte/macrophage differentiation at the expense of granulocyte differentiation (45). Microarray analysis of this GM-CSF-R-induced cell-fate switch revealed macrophage differentiation was accompanied by a robust accumulation of β-cat protein, TCF4 mRNA, and a number of β-cat/TCF4 target genes previously identified by chromatin immunoprecipitation (47), including the macrophage-lineage transcription factor, Egr-1 (48). Thus, β-cat/TCF4 signaling can direct monocytic over granulocytic cell fate in a culture system, similar to other binary cell-fate choices β-cat signaling directs throughout development and tissue homeostasis. These data may be consistent with early work showing that transduction of hematopoetic progenitor cell cultures with Wnt5a or Wnt11 expression vectors diminished the formation of macrophages, whereas Wnt11 silencing favored formation of cultures dominated by macrophages (36). Since Wnts5a and 11 are often found to inhibit β-cat signaling across numerous cell types (12), these data support the concept that β-cat signaling may be required for macrophage specification, particularly since removal of all Wnt activity from adult murine bone marrow failed to alter hematopoiesis (49).

Indeed, while GM-CSF-R-mediated upregulation of β-cat protein correlated with an inhibition of the major inhibitory kinase of β-cat, GSK3β (45), it is more likely that GM-CSF signaling is temporally and spatially uncoupled from the activation of β-cat required for macrophage specification. As discussed above, normal macrophage differentiation occurs within the interstitium of tissues, after blood monocyte recruitment, where β-cat activation is more likely to occur downstream of Wnt ligands (50). Given evidence that adult tissues are maintained by tonic Wnt signaling gradients (51–53), it seems likely that extravasating monocytes will find a Wnt-rich environment directing the monocyte-to-macrophage transition. Indeed, the spatial organization of Wnts and Wnt inhibitors may dictate where monocytes will be locally differentiated into macrophages, or even related lineages such as dendritic cells.

While macrophages across tissue types are largely transcriptionally distinct, reflecting the specialized functions of macrophages in each tissue, it is worth noting that a core group of macrophage-associated genes was recently identified (50), some of which show cross-regulation by Wnt/β-cat signaling. For example, the core macrophage factor, Bach1, can negatively regulate β-cat signaling at the level of β-cat/TCF4 interaction (54, 55). In addition, β-cat can either suppress or activate the major macrophage transcription factor CCAAT enhancer-binding protein-α (C/EBPα) (56, 57) depending on its pairing with Lef/TCF-family member isoforms. Thus, while Wnt/β-cat signaling is a plausible switch for the monocyte to macrophage transition across tissue, future mouse genetic studies with Cre-drivers that specifically target monocytes crossed with the β-cat-floxed mouse will be required to formally test this hypothesis.

### Wnt Modulation of the Immune Response

Perhaps the earliest evidence linking a Wnt ligand to inflammatory signaling came from the study of embryonic fly development, where WntD (Wnt inhibitor of Dorsal) was found to be a gene target and negative feedback regulator of Toll receptor signaling (58). Specifically, WntD overexpression inhibited the nuclear accumulation and transcriptional activity of Dorsal (an NFkB ortholog). This inhibition was independent of either the inhibitory factor of kappa β (IkB) or β-cat orthologs, suggesting a non-canonical signaling mechanism. Importantly, WntD knock-out flies were immunocompromised and showed altered expression of antimicrobial peptides as well as greater sensitivity to death by *Listeria*, phenotypes that could be rescued by the loss of Dorsal/NFkB. These data raised the possibility that Wnts found in higher organisms might be similarly upregulated by innate immunity pathways to repress the adverse effects of excessive NFkB signaling.

**Figure 3:**
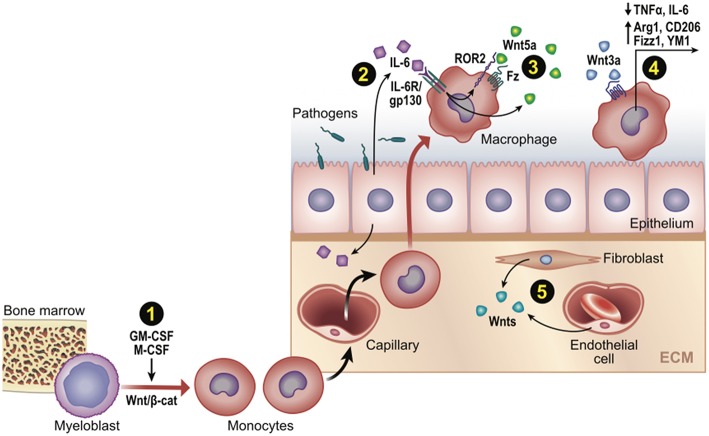
Wnt signaling in monocyte-macrophage development and function post-infection or injury. Schematic representation of Wnt-directed macrophage phenotypes. (1) Growth factors M-CSF and GM-CSF upregulate β-cat/TCF levels, which may promote differentiation of myeloblasts into monocytes in developing if not adult contexts. (2–3) Wnts can be a downstream target of core inflammatory signals (e.g., Wnt5a by IL6/NFkB), where it may promote or limit phagocytic activity. (4) Wnt/β-cat signaling often promotes an anti-inflammatory macrophage phenotype (cytokines/genes shown). (5) Various cell types may be Wnt sources (e.g., fibroblasts, endothelia, as well as epithelia and macrophages).

Although Wnt8 is thought to be the WntD ortholog in higher organisms (59), there is more evidence for other Wnts shaping macrophage immune responses, including Wnt5a (35), Wnt6 (60), and Wnt7a (39). A microarray-assisted screen of human macrophages stimulated with different mycobacterial species found that Wnt5a could be induced by TLR-NFkB signaling, where it drove the enhanced expression of Th1 cytokines, IL12, p40, and INFγ (35). Since the pro-inflammatory effect of Wnt5a appeared to signal independently of the canonical effector, β-cat (61, 62), there was much interest in cross-comparing inflammatory responses initiated by canonical β-cat-activating Wnts, particularly the widely available Wnt3a ligand. Indeed, a number of studies found that Wnt3a/β-cat signaling induced anti-inflammatory cytokine signatures, such as reduced TNFα levels (30), reduced IL6 levels (63, 64), and elevated IL4 targets [Arg1, CD206/MR, Fizz1, YM1 (27); Figure 3]. However, Wnt5a could also generate anti-inflammatory macrophage phenotypes in certain contexts (34, 38). In addition, Wnt3a/β-cat signaling could be pro-inflammatory in microglia (28) and β-cat could pair with a dsRNA nucleic acid sensor protein to induce IFN-β production downstream of *Listeria* infection (65). Thus, it appears that no uniformly distinct macrophage response may be generated between canonical (β-cat-activating) vs. non-canonical Wnts (66). Whether this reflects differences in Wnt receptor repertoires between macrophage cell lines or subsets, the contextual nature of Wnt signaling and target gene selection, or other technical issues (discussed next), remains unclear.

One complication of studying the downstream effects of particular Wnt ligands is that early “rules” for signaling gave way to exceptions, which are now seen as reflecting the highly contextual nature of this pathway. Early studies found Wnt5a to signal independently of β-cat (67), but we now know that Wnt5a can either activate or inhibit β-cat signaling depending on the presence of distinct cell-state dependent Wnt-receptors and co-receptors (15, 67). For example, when cells express Ror2, a member of the Ror-family of tyrosine kinase receptors, Wnt5a/Ror2 engagement triggers the activation of RhoA, JNK, and NFAT signaling events to control polarized cell adhesion and migration (68) [reviewed in (69)]. Conversely, in the absence of Ror2 and the presence Frizzled 4, Wnt5a can activate β-cat signaling (15). Thus, the expression and or availability of surface receptors is critical to how a particular form of Wnt will signal (12).

Another complication relevant to the study of inflammatory Wnt signaling in macrophages is that β-cat can also be robustly upregulated by the Th2-cytokine, IL4 (70). This increased β-cat is not for nuclear signaling, but rather is coupled to the cell-cell adhesion molecule, E-cadherin. Hence, conditions leading to higher levels of β-cat protein in macrophages do not necessarily mean these cells are responding to canonical Wnt signals. Lastly, other studies raise concerns that commercially available recombinant Wnt proteins bear variable inflammatory activities that cannot be antagonized by established inhibitors, such as sFRP1 (71), or are completely blocked in TLR4^−/−^ macrophages (72). Whether this is due to *bona fide* lipopolysaccharide (LPS) contamination of recombinant Wnt preparations or overlapping physiochemical properties of LPS and acylated Wnt proteins remains unclear. Thus, the use of recombinant Wnts to interrogate inflammatory pathways should be considered judiciously and verified using forced expression, knock-down or targeted gene loss approaches.

Using these latter approaches, a number of studies clearly confirm roles for β-cat in shaping macrophage activation states. Pro-inflammatory macrophages, originating from newly recruited monocytes, are primed by TLR agonists of bacterial or viral nature and characterized by the production of pro-inflammatory cytokines, high phagocytosis activity and production of reactive oxygen species (ROS). These inflammatory macrophages not only clear pathogens, but also produce tissue damage. Upon phagocytosis of dead and apoptotic cells, these inflammatory monocyte-derived macrophages can convert into pro-repair cells, characterized by increased expression of IL10, Arg1, Tgm2 [reviewed in (3)]. Two recent studies found that tumor-derived Wnt ligands can stimulate an anti-inflammatory tumor-associated-macrophage phenotype via β-cat signaling, which contributes to tumor growth, migration, metastasis, and immunosuppression via elevated expression of *Arg1, Sti1, IL10*, and decreased expression of *IL1b*, respectively [(29, 32); Figure 3].

Perhaps the best examples of β-cat signaling driving anti-inflammatory phenotypes have been focused on the dendritic subset of monocyte-derived cells, using *Cd11c*^Cre^ targeted genetic approaches to activate or inhibit β-cat signaling in mice (73–75). These studies independently confirm roles for β-cat in the upregulation of key anti-inflammatory mediators, such as IL-10 and TGFβ, as well as tolerogenic T-cell behaviors relevant to tumor immunosuppression and inflammatory bowel disease (76, 77). Investigation into how distinct macrophage populations contribute to fibrosis revealed that genetic removal of β-cat from either *Csf1r*- or *Cd11c*-Cre expressing macrophages attenuates fibrosis in the both kidney (26) or lung injury models (78), through limiting the abundance/maturation of monocyte-recruited macrophages. The former study confirmed that *Csfr1*-Cre; βcat-floxed mice also show an anti-inflammatory profile (26). While macrophages are weak antigen presenting cells, showing less capacity to travel to lymph nodes for antigen presentation to T-cells than dendritic cells, these data nonetheless raise the possibility that orchestration of anti-tumor immunity and fibrosis resolution may depend on modulated β-cat signaling in both macrophage and dendritic cells. Since most tissue macrophages also express Cd11c and are therefore targeted using this recombinant genetic approach, future studies are required to delineate the precise roles of these subpopulations of the macrophage lineage.

### Wnt Signals Control Specific Macrophage Functions

#### Phagocytosis

Evidence from *Drosophila* suggests that Wnt signaling may control a cell's response to different pathogens. For example, *WntD* mutants were more susceptible to the intracellular pathogen, *L. monocytogenes*, yet more resistant to the extracellular pathogen, *S. pneumoniae* (43). The loss of WntD is thought to lead to enhanced phagocytic activity and a shift in anti-microbial peptide that favors fast killing of an extracellular pathogen, but at the expense of inadequately controlling cytoplasmic access of an intracellular pathogen. There is some evidence confirming Wnt signals can also affect macrophage phagocytosis in mice, but molecular details remain unclear. In a macrophage cell line, recombinant Wnt5a stimulated phagocytosis of bacteria through a β-cat-independent signaling mechanism (37), and recombinant Wnt7a, but not Wnt1, inhibited phagocytosis in spleen monocyte-derived macrophages, correlating with reduced expression of surface molecules implicated in phagocytic uptake (e.g., CD14, CD11b, CD163, CD206) (39). Importantly, *Wnt7a*^−/−^ mouse monocyte-derived macrophages showed elevated surface levels of CD11b, which could be reduced by adding back recombinant Wnt7a. Wnt signaling also plays a role in macrophagic phagocytosis in tissue development. Mice deficient in Lrp5, a critical Wnt co-receptor, displayed persistent embryonic eye vascularization due to a failure of macrophage-induced endothelial cell apoptosis (79). Although the precise mechanism is unclear, it is worth noting that in *C. elegans*, machinery required for the recognition and engulfment of dead cells can also promote apoptosis when caspase activity is compromised. Thus, Wnt-regulated phagocytes may provide a key backup mechanism for removing superfluous cells (80). Lastly, Lrp5 is also important for macrophage phagocytosis and clearance of lipids (e.g., low density lipoproteins), as *Lrp5*^−/−^ mice are prone to development of atherosclerosis when fed a high fat diet (81, 82). Thus, Wnt receptors and downstream signaling contribute to macrophage phagocytosis in a variety of developmental and disease contexts.

#### Wnts and Macrophage Adhesion, Migration, and Tissue Recruitment

As Wnt signaling has been linked to numerous cell migratory events from gastrulation to neural crest cell dissemination [reviewed in (83)], it is attractive to consider this signaling module might be conserved in macrophages. Activation of β-cat signaling via Wnt3a (conditioned media) in monocytes (isolated from healthy donor peripheral blood) limited their migration through an endothelial layer of human dermal microvascular endothelial cells, while not affecting their motile capacity or ability to adhere to endothelial cells (31). In other contexts, Wnt co-receptor Lrp5 signaling contributed positively to macrophage motility (84, 85). Whether Wnts are locally secreted by an activated endothelium to modulate the recruitment of monocytes to move across tissue capillary beds remains unknown. Evidence CD11b (an integrin required for tissue migration) is upregulated on the surface of *Wnt7a*^−/−^ mouse bone marrow-derived macrophages, showing enhanced tissue recruitment relative to controls (39), whereas CD11b is reduced on the surface of *Csfr1*^Cre^; β-cat null cells, showing less tissue recruitment than controls (26), strongly suggest that β-cat signaling in macrophages can regulate their entry and/or retention within tissues. Evidence also supports the concept that β-cat signaling in stromal cell populations beneath the vascular bed may direct the expression of chemokines that promote macrophage infiltration [e.g., via Cxcl12 (86)].

## Macrophages as a Source of Wnts to Which Parenchymal Cells Respond

Given that macrophages play critical paracrine functions during tissue development and injury repair [reviewed in (87)], it is probably not surprising that numerous studies reveal macrophages as a source of morphogenic Wnt signals to guide stem cell behaviors during these processes.

### Tissue Development and Renewal

Skin macrophages can drive the cyclical activation of hair follicle stem cells through an apoptosis-associated release of Wnts7b and 10a within the hair follicle niche (40). Since dermal macrophages have long been appreciated to resorb collagen during hair follicle regression (88–90), it is attractive to consider that the same cell-type which drives matrix scavenging can also dispatch regenerative ligands for the next round of hair growth. Similarly, the mammary gland epithelium forms post-natally during adolescence in a process called branching morphogenesis. For this to occur, epithelial progenitors must bifurcate (divide) and lengthen the terminal end bud into the fat pad, a process that requires macrophage-mediated remodeling of the collagen-rich matrix (91, 92). Recent evidence shows mammary stem cell expression of the Notch ligand, Dll1, activates Notch signaling in macrophages to stimulate Wnt secretion (*Wnt3, 10a*, and *16*) (25). As with dermal macrophages in hair follicle cycling, mammary macrophages coordinate the matrix remodeling required for branching morphogenesis with the delivery of Wnt ligands to expand epithelial progenitors. In the gastrointestinal system, gut macrophages play an important role in signaling an overall readiness of the intestinal epithelium to sample antigens from the gut lumen. Anti-CSF1R approaches that effectively ablate macrophages resulted in perturbed intestinal stem cell abundance and subsequent lineage choices, such as elevated goblet cells and a reduction in M-cells along Peyer's patches, which would limit antigen sampling by sub-epithelial macrophages (93). Further studies of tissue-resident macrophages utilizing Wnts to maintain homeostasis in other adult tissues may reveal further similarities or organ-specific roles.

### Repair After Injury

Perhaps the best evidence macrophages are a meaningful source of Wnts come from murine tissue-injury models. Conditional deletion of *Wnt7b* in Csf1R-expressing macrophages inhibited kidney repair after ischemia/reperfusion injury (41). During chronic liver injury, macrophage engulfment of hepatocyte debris induced Wnt3a expression, which stimulated β-cat signaling in nearby hepatocyte progenitors and their differentiation into hepatocytes (24). Critically, when all active Wnt secretion is prevented in liver macrophages using the *Wls*^Cre^ genetic approach, the normal Wnt gradient that controls liver hepatocyte zonation remains intact, yet liver regeneration after partial hepatectomy was impaired (94). Thus, in the liver, macrophage-derived Wnts are not important for normal tissue homeostasis, but rather for repair after injury. While the injury signals that promote the upregulation of macrophage-derived Wnts remain poorly defined, one recent study found that *Cd11c*-expressing lung macrophages promote epithelial proliferation and repair post-injury through a Trefoil factor 2-dependent mechanism that leads to the upregulation of Wnts4 and 16 (33). Lastly, it is worth noting that macrophages associated with lung disease can manifest high levels of the Wnt pathway agonist, R-spondin-3 (*RSPO3*) (95). Therefore, it appears that macrophage-derived Wnts, or factors that synergize with Wnts, may be a general feature of the tissue injury and repair cycle. Indeed, it may be parsimonious for the cell-type required to remove cellular debris and remodel basement membranes to also bring factors which stimulate renewal of the injured tissue.

There is also evidence supporting the concept that adult tissues may display tonic expression of Wnts, which may shape the identity and activity of macrophage sub-populations. For example, using single cell RNA sequencing of human fibrotic lungs, a small group of highly expressed Wnt ligands were apparently restricted to specific cell types, such as *WNT2* in fibroblasts, *WNT7b* in alveolar type 2 and club cells, and *WNT7a and WNT3a* in type 1 cells (95). If Wnt proteins are secreted from basolateral rather than luminal apical surfaces (as is the case for most growth factors/morphogens), we speculate that during normal tissue homeostasis, interstitial macrophages may be constitutively subjected to a high Wnt environment, whereas alveolar macrophages may occupy a Wnt-free lumenal environment, which may shape their distinct transcriptional profiles (7). Intriguingly, RNA-sequencing of recently characterized population of pro-fibrotic, recruited monocyte-derived alveolar macrophages revealed altered expression of Wnt/β-cat pathway components and target genes (Lam, Gottardi, and Misharin, unpublished). Since deletion of β-cat from CD11c+ alveolar macrophages reduced the number of recruited monocyte-derived alveolar macrophages in the bleomycin model of murine fibrosis (78), we speculate that tissue injury, which may destroy the normal compartmental separation of Wnt-abundant from Wnt-free environments and/or upregulate Wnt expression, may be responsible for the aberrant differentiation state of pro-fibrotic alveolar macrophages.

### Cancer Progression

It is well-established macrophages can both drive tumorigenesis and shape anti-tumor immunity [reviewed in (1, 96)]. An early example of the former came from multiphoton intravital imaging of a murine model of breast cancer, which showed epithelial expression of Csf1 promotes the infiltration of Csfr1+ macrophages, while macrophages act as a source of EGF, leading to a mutually reinforcing paracrine signaling loop of CSF-1 and EGF ligands between macrophages and tumor cells that drive tumor invasion and metastasis (97, 98). More recent studies support the idea that tumor associated macrophages may produce Wnts that contribute to tumor cell invasiveness (99–102), although Wnts were not formally removed in these studies. Importantly, genetic removal of *Wnt*7b from myeloid cells using an inducible *Csfr1*^Cre^ approach appeared sufficient to reduce mammary tumor burden through inhibition of angiogenesis, possibly due to reduced VEGFa expression in vascular endothelial cells (42).

While evidence for Wnt-expression in macrophages relevant to tissue development, repair, and cancer progression appears clear, it should be noted that the absolute abundance of Wnt expression by macrophages is substantially less than other cell types within the same tissues, as evidenced by various single-cell RNA sequencing data sets that have become available (95) and by studies employing quantitative PCR methods (32, 101). While formal genetic evidence for macrophage-derived Wnts using currently available Cre-drivers exists (42, 103), it is important to recognize that Cre-drivers are often more broadly expressed than appreciated, particularly under injury-induced conditions, and should be considered when interpreting results from such models.

## Summary

Growing knowledge at the intersection of macrophage biology and Wnt signaling reveals multiple roles for Wnt signals coming from and within the monocyte-macrophage lineage. Wnt signals received by macrophages are important for their various phagocytic roles, such as modulating the immune response in the setting of infection, tissue repair after injury, malignancy detection, and progression. Conversely, macrophages can be a source of Wnt signals critical for tissue morphogenesis and healing after injury. A key future direction will be to understand how tissue-specific macrophage identities are shaped by their local environment. Since Wnts have been classically shown to act locally, typically only a few cell diameters away from the source of Wnt secretion (23) evidence that adult tissues are maintained by local Wnt signaling niches (51–53) raise the intriguing possibility that such tissue-specific Wnt patterns may contribute to the variety of macrophage-type and behaviors critical to organ development and homeostasis.

## Author Contributions

All authors listed have made a substantial, direct and intellectual contribution to the work, and approved it for publication.

### Conflict of Interest Statement

The authors declare that the research was conducted in the absence of any commercial or financial relationships that could be construed as a potential conflict of interest.
